# Here Today, Here Tomorrow? Urinary Concentrations of Parabens over Time

**DOI:** 10.1289/ehp.120-a437b

**Published:** 2012-11-01

**Authors:** Tanya Tillett

**Affiliations:** Tanya Tillett, MA, of Durham, NC, is a staff writer/editor for *EHP*. She has been on the *EHP* staff since 2000 and has represented the journal at national and international conferences.

Parabens are antimicrobial preservatives added to foods, pharmaceuticals, and personal care products. These compounds have been detected in 92% of a representative sample of the U.S. population. The two most commonly used forms—methyl paraben (MP) and propyl paraben (PP)—are classified as “generally recognized as safe” by the U.S. Food and Drug Administration, although there is some animal and human evidence these chemicals may be endocrine disruptors. The ubiquity of paraben exposure and the potential for adverse health effects prompted investigators to study how exposure varies over time in men and women [*EHP* 120(11):1538–1543; Smith et al.].

Between August 2005 and November 2010 the research team collected an average of 4 spot urine samples each from 245 male and 408 female patients at the Fertility Center at Massachusetts General Hospital in Boston. Samples were collected upon recruitment for the study, at followup visits during infertility treatments, and during pregnancy, and were analyzed for concentrations of MP, PP, and the less widely used butyl paraben (BP).

MP and PP, which are often used in combination, were detected in more than 95% of samples, with a high correlation between the two suggesting a common source of exposure. MP and PP concentrations were more than 4 times higher in women than in men and more than 3 times higher in African Americans than in Caucasians. BP was detected in more than twice as many women (74%) as men (36%), and concentrations were more than 4 times higher in women than in men but less variable between Caucasians and African Americans. These results could reflect differences between men and women and between African Americans and Caucasians in the use of products containing parabens. They also could reflect pharmacokinetic differences.

Women showed more temporal variation of urinary paraben concentrations than men, possibly reflecting changes in their use of personal care products over time. Those women who became pregnant during the study period generally had lower urinary paraben concentrations during pregnancy than before, with evidence suggesting that concentrations decreased with each additional week of pregnancy. This, too, could reflect changes in foods eaten or products or medications used during pregnancy.

Nevertheless, the relative stability of individuals’ urinary paraben concentrations over time suggests that a single urine sample could provide a reasonably representative snapshot of an individual’s exposure to parabens over the course of several months. Likewise, a single urine sample collected during pregnancy could reasonably predict exposure throughout pregnancy. However, results were based on a population that was mostly Caucasian and well educated, and their generalizability to other demographic groups is uncertain.

